# Police Officers’ Ability in Recognizing Relevant Mental Health Conditions

**DOI:** 10.3389/fpsyg.2021.727341

**Published:** 2021-09-17

**Authors:** Linus Wittmann, Gunter Groen, Petra Hampel, Ronja Petersen, Astrid Jörns-Presentati

**Affiliations:** ^1^Department of Health Psychology and Health Education, Europa-Universität Flensburg, Flensburg, Germany; ^2^Department of Social Work, University of Applied Sciences Hamburg, Hamburg, Germany; ^3^Hamburg Police Academy, Hamburg, Germany

**Keywords:** individuals with mental disorders, training, mental health conditions, mental health literacy, police

## Abstract

The recognition of certain mental health conditions is important as this requires police officers to communicate and behave in an adjusted manner with affected individuals. The objective of the present study was to test police officers’ knowledge about mental health symptoms as a component of their mental health literacy (MHL) and to examine if police officers’ perceived knowledge corresponds with their actual knowledge. A questionnaire was used to assess for MHL representing mental health conditions which occur frequently in police requests (schizophrenia, bipolar disorder, depression, post-traumatic stress disorders, and emotionally unstable personality disorder). Furthermore, the questionnaire assessed the frequency of police requests, the officers’ perceived knowledge regarding mental disorders and their sense of feeling sufficiently trained to deal with these kinds of requests. Eighty-two police officers participated in the study. Police officers’ actual knowledge about mental health conditions did not correspond with their perceived knowledge. Participants revealed a moderately high level of overall knowledge which differed with regard to symptoms of each of the five mental health conditions. The mental status of a paranoid schizophrenia was best identified by the police officers and the majority correctly allocated the symptoms. Post-traumatic stress disorders and manic episodes were only identified by a minority of police offers. Police training geared to prepare for requests involving individuals with mental disorders should expand this limited knowledge transfer and focus on a broader variety of mental health conditions that police officers frequently encounter in requests.

## Introduction

There is a large body of research examining interactions between police forces and individuals with a mental health condition (Vigours and Quy, 2017). Previous research examined the frequency (Livingston, 2016), circumstances (Charette et al., 2014), and the subjective experience of affected individuals (Livingston et al., 2014b; Soares and Pinto da Costa, 2019) as well as involved police officers in these interactions (Wittmann et al., 2021c). Additionally, there are studies suggesting that police officers regularly encounter individuals diagnosed with schizophrenia, bipolar disorder, depression, post-traumatic stress disorders (PTSD) or emotionally unstable personality disorders (EUPD; Resnick et al., 2000; Kesic et al., 2013; Jones and Thomas, 2019; O’Neal, 2019). The crisis intervention team training (CIT) program proposes that police officers require knowledge about different kinds of mental health conditions in order to correctly detect them in real life situations (Watson and Fulambarker, 2012; CIT International, 2019). It is thought that the ability to recognize and identify certain mental health conditions enables police officers to adjust their communication strategies and behavior with a focus on procedural justice, empathy and patience to prevent the risk of escalating violence and traumatic experiences (Desmarais et al., 2014; Livingston et al., 2014a; Wittmann et al., 2021b). Knowledge about and recognition of mental health conditions is one component of the mental health literacy (MHL) concept. MHL was first described by Jorm et al. (1997) and builds upon the concept of health literacy (Nutbeam, 2000; World Health Organization, 2013). Components of MHL include (a) the ability to recognize specific disorders or psychological distress; (b) knowledge and beliefs about risk factors and causes; (c) knowledge and beliefs about self-help interventions; (d) knowledge and beliefs about available professional help; (e) attitudes which facilitate recognition and help-seeking; and (f) knowledge of how to seek mental health information (Jorm, 2000).

In their regular practice, German police officers are not expected to be able to identify the wide range of symptoms indicating potential mental health symptoms, but they need to be able to recognize certain mental health conditions. However, there is evidence that police officers only identify mental health problems in 50% of the encounters they have with individuals with mental health issues (McKinnon and Grubin, 2013). To this date, there is a paucity of research examining whether German police officers are capable of correctly identifying individuals suffering from poor mental health. The aim of the present study was therefore to test German police officers’ knowledge about mental health symptoms and draw conclusions about the extent to which they are mental health literate. In addition, we tested if police officers’ perceived knowledge corresponded with their actual knowledge.

## Materials and Methods

### Materials

Two of the authors, a clinical psychologist and a police officer, constructed the questionnaire to assess for MHL based on the criteria of the International Classification of Diseases and related Health Problems 10 (ICD-10; WHO, 2015). We chose five mental health disorders which occur frequently in police requests: schizophrenia, bipolar disorder, depression, PTSD, and EUPD (Resnick et al., 2000; Kesic et al., 2013; Jones and Thomas, 2019; O’Neal, 2019). The questionnaire contained the description of 17 symptoms using clinical terminology (e.g., paranoid delusions) which participants had to assign to one of five different mental health conditions. We used the terminology of the ICD-10 to label symptoms and mental health disorders. To meet the requirements of a practical oriented approach certain symptoms were selected which are (1) characteristic for the respective diagnosis and (2) salient in the communication or the interaction with an individual. Furthermore, the questionnaire inquired about age, gender, and years of service. The frequency of police requests involving individuals with mental disorders was assessed on a five-point Likert scale from several times a day to never before. Police officers were also asked to evaluate their perceived knowledge regarding mental disorders and sense of feeling sufficiently trained to deal with these kinds of requests on a five-point Likert scale (1=*very poor* to 5=*very good*). To assess the affective component of these interactions, participants were asked a single choice question measuring if they felt predominantly safe, tensed, uneasy or overwhelmed in requests involving individuals experiencing a mental health condition.

### Procedure

The stratified data collection was performed over two weeks in June 2020. Participating frontline police officers were recruited in a large German city. In order to achieve a representative sample of the city, five police departments located in the center and in the western, eastern, southern, and northern districts were chosen. Pervious research showed that police interactions with individuals with mental illnesses are not equally allocated in neighborhoods (Krishan et al., 2014). Therefore, we deemed this approach necessary as different police departments were located in neighborhoods with potentially differing frequencies in police request involving mental disorders. A paper-pencil survey was used as the most practical approach to collect data in the field. The questionnaires were distributed to the selected police departments by a police officer student and handed to the post office boxes of all frontline police officers of the respective police department. Additionally, the superintendents of each unit were personally informed about the study. Based on preliminary considerations of the manpower of each department it was deemed sufficient to provide each department with *N*=40 questionnaires resulting in a potential sample of *N*=200. Additionally, participants were provided with a letter informing about the study and an informed consent form. Participants were able to contact a researcher for any questions regarding the study. Questionnaires and informed consent forms were collected separately in two different sealed boxes over a period of two weeks. This period was chosen in order to increase the response rate in case some police officers would be on vacation or attend training. The sealed boxes were only opened by the researchers to maintain anonymity and confidentiality. The study was reviewed by the respective police academy. This included the involvement of the staff council. In addition, the research is in accordance with the Declaration of Helsinki.

## Results

### Participants

A total of *N*=82 (response rate=41%) police officers participated (33 female, 40.2%) in the study. The age ranged most frequent from 26 to 35years (*n*=26, 32%) and most police officers (*n*=24, 29%) had spent less than 6years in service. Most participants (*n*=51, 62.2%) experienced police requests with individuals with mental disorders several times per month. The majority (*n*=52; 63.4%) estimated their knowledge in the category as moderate (see Table 1). The majority of participants (*n*=47; 57.3%) reported they had felt tense during interactions with individuals with mental disorders, about a quarter (24.4%) of the participants felt safe, 14 (17.1%) participants felt uncomfortable, and one (1.2%) participant felt overwhelmed.

**Table 1:** Frequency distribution of age, years spent in service, frequency of requests involving individuals with a mental disorder, and perceived knowledge.

Age	18–25	26–35	36–45	46–55	>56
*N* (%)	17 (21%)	26 (32%)	25 (30%)	11 (13%)	3 (4%)
Years spent in service	<5	6–15	16–25	26–34	>35
*N* (%)	24 (29%)	23 (28%)	23 (28%)	7 (9%)	5 (6%)
Frequency of requests	Multiple times per day	Multiple times per week	Multiple times per month	Less than once in a month	Never before
*N* (%)	51 (62.2%)	23 (28%)	6 (7.3%)	2 (2.4%)	–
Perceived knowledge	Very good	Good	Moderate	Poor	Very poor
*N* (%)	2 (2.4%)	21 (25.6%)	52 (63.4%)	7 (8.5%)	–
Sense of feeling trained*N* (%)	–	13 (15.9%)	38 (46.3%)	27 (32.9%)	4 (4.9%)

### Overall Mental Health Literacy

In order to test if police officers’ actual knowledge corresponded with their perceived knowledge we calculated a knowledge score. This score represents how many symptoms were correctly assigned to one of the five mental health conditions resulting in a possible range from 0 (*all incorrect*) to 17 (*all correct*). This score was available for *n*=52 participants due to missing values. Participants average knowledge score was *M*=10.94 (SD=2.26). A Spearman rank correlation did not show a significant association between the police officers’ actual knowledge and subjective knowledge.

### Paranoid Schizophrenia

Fifty-one (62.2%) participants correctly identified the three key symptoms of paranoid schizophrenia (see Table 2). Paranoid delusions (*n*=67; 81.7%), bizarre delusions (*n*=63; 76.8%), and acoustic (auditory) hallucinations (*n*=67; 81.7%) were to the same extent correctly identified as symptoms of a paranoid schizophrenia.

### Emotionally Unstable Personality Disorder (Borderline Personality Disorder)

No participant correctly identified all three symptoms of an EUPD. Self-harming behaviors, including suicide gestures, were correctly assigned to EUPD by 54 participants (65.9%). Outbursts of emotions due to emotional liability were identified by 33 (40.2%) participants as a symptom of EUPD and seven (8.5%) participants assigned chronic feelings of emptiness to the disorder.

### Post-traumatic Stress Disorder

Twelve participants (14.6%) correctly identified all four symptoms of a PTSD. Flash backs were identified by the majority of the police officers (*n*=75; 91.5%) as a specific PTSD symptom. Sleeping disturbances (*n*=56; 68.3%) and enhanced startle reactions (*n*=47; 57.3%) were frequently identified as a PTSD symptom, whereas avoidance behaviors were only identified by one in four participants (*n*=20; 24.4%).

### Manic Episode

All symptoms of a manic episodes were only identified by 14 (17.1%) participants. Elevated mood (*n*=48; 58.5%) and inflated self-esteem and grandiose ideas (*n*=46, 56.1%) were more frequently perceived as qualifiers for a manic episode than overactivity (*n*=37; 45.1%) and reckless behavior (*n*=32; 39%).

### Depressive Episode

Thirty-six participants (*n*=36; 44%) identified all four symptoms of a depressive episode. Reduction in energy (*n*=68; 83%) and lowering of mood 68 (83%) were identified more frequently as depressive symptoms compared to suicidal thoughts (*n*=50; 61%) and sleep disturbances (*n*=56; 68.3%; Table 2).

**Table 2:** Correct and false allocations of the 17 symptoms and five mental health conditions.

	Correct, *n* (%)	False, *n* (%)	Missing, *n* (%)
Paranoid schizophrenia	51 (62.2%)	22 (26.8%)	9 (11%)
Paranoid delusions	67 (81.7%)	10 (12.2%)	5 (6.1%)
Bizarre delusions	63 (76.8%)	15 (18.3%)	4 (4.9%)
Acoustic hallucinations	67 (81.7%)	8 (9.8%)	7 (8.5%)
Emotionally unstable personality disorder (EUBD)	–	62 (75.6%)	20 (24.4%)
Liability to outbursts of emotion	33 (40.2%)	40 (48.8%)	9 (11%)
Chronic feelings of emptiness	7 (8.5%)	68 (82.9%)	7 (8.5%)
Self-destructive behavior, including suicide gestures	54 (65.9%)	10 (12.2%)	18 (22%)
Post-traumatic stress disorder (PTSD)	12 (14.6%)	51 (62.2%)	19 (23.2%)
Avoidance behavior	20 (24.4%)	50 (61%)	12 (14.6%)
Flashbacks	75 (91.5%)	5 (6.1%)	2 (2.4%)
Enhanced startle reactions	47 (57.3%)	32 (39%)	3 (3.7%)
Sleeping disturbances	56 (68.3%)	11 (13.4%)	15 (18.3)
Manic episode	14 (17.1%)	49 (59.7%)	19 (23.2%)
Elevated mood	48 (58.5%)	27 (33%)	7 (8.5%)
Overactivity	37 (45.1%)	36 (43.9)	9 (11.0)
Reckless behavior	32 (39%)	43 (52.4)	7 (8.5%)
Inflated self-esteem and grandiose ideas	46 (56.1%)	30 (36.6%)	6 (7.3%)
Depressive episode	36 (44%)	23 (28%)	23 (28%)
Reduction in energy	68 (83%)	7 (8.5%)	7 (8.5%)
Lowering of mood	68 (83%)	3 (3.6%)	11 (13.4%)
Suicidal thoughts	50 (61%)	13 (15.8)	19 (23.2%)
Sleep disturbances	56 (68.3%)	11 (13.4%)	15 (18.3%)

## Discussion

The aim of the present study was to test police officers’ MHL with regard to their knowledge of relevant mental health conditions. Our first finding is that police officers have a moderate knowledge of the symptoms of mental illnesses that they encounter more frequently during police requests. Second, police officers revealed a differentiated knowledge about mental disorders in general: A diagnosis of paranoid schizophrenia was identified most frequently by participants (62%) and the majority correctly allocated the respective symptoms to the disorder. A depressive episode was the second best identified mental health condition in our sample (44%). This is in line with research demonstrating that higher rates of individuals diagnosed with schizophrenia or depression compared to other mental disorders come in contact with police (Kesic et al., 2013; McKinnon and Grubin, 2013). Additionally, McKinnon and Grubin (2013) showed that police officers detected symptoms of schizophrenia more commonly compared to other mentals health conditions (e.g., intellectual disabilties). However, one could argue that German police training focuses specifically on schizophrenia and therefore police officers present with a broader knowledge (Wundsam et al., 2007; Wittmann et al., 2021a). In contrast, PTSD (15%) and manic episodes (17%) were only identified by a minority of police offers and EUPD by none of the participants. This finding is unexpected, as police encounters with individuals diagnosed with bipolar disorders and EUPD occur relatively often in police requests (Gandhi et al., 2001; Kesic et al., 2013). There are two possible explanations for this result. First, the low detection rates may be due to the symptoms we selected that police officers were not familiar with. Second, it is possible that police officers knew the symptoms but were not able to assign these to the corresponding mental health condition. Thus, we suggest that police training that sufficiently prepares police officers for requests involving individuals with mental disorders should focus on expanding police officers’ knowledge transfer with a focus on a broader variety of mental health conditions that occur frequently in police requests.

### Limitations

The present study has certain limitations with regard to the measurement and sample used and these should be considered when interpreting the results. First, the questionnaire was constructed in order to test for certain symptoms of selected mental disorders. When describing symptoms we used a clinical terminology offered by the ICD-10 (WHO, 2015). It is possible that participants were not familiar with these terms which could have resulted in unanswered questions or guessing. Future research should examine which terminology police officers use in their daily routines to describe signs of mental symptoms. Second, we only used a limited number of three to four of the symptoms listed in the ICD-10 to represent each mental disorder, which may have limited the validity of the questionnaire. Additionally, some of the symptoms could exist in multiple types of mental illness even if the ICD-10 suggest a clear distinction. The rationale behind this, was our assumption that police officers in the field may also only use selected indicators to screen for mental health conditions instead of conducting comprehensive diagnostics. Third, the present study did not consider co-occurring mental disorders, however there is a certain amount of individuals that experience multiple diagnoses (Jones and Thomas, 2019). Fourth, the present study comprised a relatively small sample size. We cannot rule out a selection bias, however another recent German study showed comparable sociodemographic characteristics of the police officers sample (Wittmann et al., 2021c). In addition, the sample size is sufficient considering the explorative character of the study. Future research should apply established MHL scales in the field of police work (O’Connor and Casey, 2015) with larger and more representative samples. Further research is needed that examines police officers’ abilities to detect if an individual is experiencing active symptoms of a mental health condition in real life encounters as our results are limited to tested knowledge.

## Conclusion

The results indicate that German police officers have a moderately high overall but distinct knowledge regarding symptoms of specific mental health conditions. Symptoms of paranoid schizophrenia were correctly identified by the majority of the police officers. Police training that aim to prepare for requests involving individuals with mental disorders (e.g., police use of force training) should also include other mental health conditions known to be relevant for police encounters with individuals with mental health problems, such as EUPD, PTSD, manic episodes and depressive episodes. Future research should examine MHL in police officers with more established measures and in more representative samples.

## Data Availability Statement

The original contributions presented in the study are included in the article/supplementary material, further inquiries can be directed to the corresponding author.

## Ethics Statement

Ethical review and approval was not required for the study on human participants in accordance with the local legislation and institutional requirements. The participants provided their written informed consent to participate in this study.

## Author Contributions

LW and RP collected the data and performed the analyses. LW, AJ-P, and GG wrote the original draft. LW, AJ-P, GG, and PH contributed to the article. All authors contributed to the article and approved the submitted version.

## Funding

We acknowledge financial support for Article Publication Charges by Land Schleswig-Holstein within the funding programme Open Access Publikationsfonds.

## Conflict of Interest

The authors declare that the research was conducted in the absence of any commercial or financial relationships that could be construed as a potential conflict of interest.

## Publisher’s Note

All claims expressed in this article are solely those of the authors and do not necessarily represent those of their affiliated organizations, or those of the publisher, the editors and the reviewers. Any product that may be evaluated in this article, or claim that may be made by its manufacturer, is not guaranteed or endorsed by the publisher.
